# Zero burden multi night monitoring with AI enabled technology reduces obstructive sleep apnea misdiagnosis

**DOI:** 10.1038/s41746-026-02914-w

**Published:** 2026-06-16

**Authors:** Bastien Lechat, Simon Proctor, Jack Manners, Kelly A. Loffler, Alison Leviton, Barbara Toson, Daniel Vena, Andrew Vakulin, Josh Fitton, Robert J Adams, Sutapa Mukherjee, Danny J. Eckert

**Affiliations:** 1 Flinders Health and Medical Research Institute (FHMRI) Sleep Health, Flinders University, Adelaide, SA Australia; 2 Department of Respiratory Sleep Medicine & Ventilation, Flinders Medical Centre, Southern Adelaide Local Health Network, SA Health, Adelaide, SA Australia; 3 Flinders Clinical Trials, Flinders University, Adelaide, SA Australia; 4 Division of Sleep and Circadian Disorders, Brigham and Women’s Hospital and Harvard Medical School, Boston, MA USA

**Keywords:** Diseases, Health care, Medical research

## Abstract

Multi-night measurement of obstructive sleep apnea (OSA) using AI-enabled technologies could reduce misdiagnosis rates from night-to-night variation. However, prospective feasibility and effectiveness of multi-night assessment in clinical populations remains uninvestigated. In this study, 100 people with suspected OSA were recruited to receive an under-the-mattress Withings Sleep Analyzer (WSA) to quantify in-home sleep for 3 months alongside single-night polysomnography. The primary outcome was feasibility of novel technology to estimate the apnea-hypopnea-index (AHI), defined as ≥14 nights of AHI recordings within the first month. Secondary outcomes included diagnostic classification of moderate-to-severe OSA (AHI ≥ 15 events/h). 92 participants (53±15yo; BMI = 31 ± 7 kg/m^2^; 48 females) were included in the final analyses. Eighty-five (92%) had ≥14 nights of valid AHI measurements in the first monitoring month. WSA had specificity of 85% and sensitivity of 77%, and overall F1-score of 80% to detect OSA versus polysomnography. Participants with OSA on WSA but not polysomnography had higher night-to-night variability in OSA severity, and many had minimal supine sleep and short sleep duration during polysomnography (-97 [-183, -11]min, *p* value = 0.027). Multi-night zero-burden AI monitoring of OSA is feasible and could identify patients at risk of single-night polysomnography misdiagnosis, including those with high night-to-night variability, short sleep, and minimal supine sleep during polysomnography.

## Introduction

Obstructive sleep apnea (OSA) is a common chronic sleep disorder characterized by repetitive complete or partial collapse of the upper airway during sleep. Approximately 1 billion adults globally are estimated to have at least mild OSA^1,2^, and OSA prevalence is expected to rise over the next 30 years^3,4^. Untreated OSA is associated with poor cardio-metabolic health, reduced quality of life and wellbeing, and has major impacts on road and workplace safety^5–11^.

Recent studies that have used simplified, artificial-intelligence-based monitoring technologies have highlighted substantial night-to-night variability in OSA severity^2,12,13^. These findings raise serious concerns about diagnostic accuracy. Indeed, at least 20% of people with suspected OSA may be misdiagnosed with the conventional single-night polysomnography-derived apnea-hypopnea index (AHI)^2,12,14,15^. In addition, up to 50% may have their OSA severity miscategorised, which is especially true for those classified as mild to moderate OSA with one night polysomnography^2,12^. Similar misdiagnosis rates are observed with type 1 and type 2 sleep studies, as well as with simplified wearables and non-wearable technologies^12^. In addition to high misdiagnosis rates, emerging evidence indicates that high variability in OSA severity from one night to the next is an important predictor of poor health outcomes, including hypertension and cardiovascular disease^16–21^, independent of OSA severity. Thus, multi-night information on OSA severity may provide novel clinical insight not currently available with single-night polysomnography.

The physiological mechanisms underpinning night-to-night variability in OSA severity are not well understood. Some studies have shown that body and head position during sleep can contribute to OSA severity and variability^22–24^. A recent large observational study has shown that there are seasonal and weekend-weekday differences in OSA severity^25,26^, perhaps mediated, at least in part, by increased sleep irregularity^27,28^. Other environmental factors such as air pollution and temperature may also impact night-to-night variability in OSA severity^4,29–31^. Demographic and behavioural factors (e.g., smoking, alcohol intake, age) also have the potential to contribute to increased night-to-night variability in OSA severity^22,32^. Accordingly, multi-night measurement of OSA severity in a naturalistic, real-world sleep environment may provide a more accurate OSA diagnosis and help reduce high disease misclassification rates with single-night polysomnography.

Simplified technologies such as mandibular movement-based sensors^33^, chest sensors^34^, forehead devices^35^, finger-worn devices^36,37^, mattress sensors^38,39^, RADAR based technologies^32^ and others (see Pinilla et al. ^40^ for a recent review) provide considerable promise to estimate OSA severity non-invasively. These devices have ~50 to 80% sensitivity and specificity to classify moderate-to-severe OSA based on the estimated AHI versus polysomnography-based AHI classification^40^. Many have already been implemented in multi-night research studies^22,32,41^. However, most recording periods were likely too short ( < 10 nights) to accurately assess OSA variability. One recent study undertook a retrospective analysis in 56 patients with suspected OSA^42^. A total of 22 sleep experts retrospectively evaluated diagnostic data twice, blinded, with single in-laboratory respiratory polygraphy only and once with the additional information of 14 nights of pulse oximetry at home. For 57% of patients with suspected OSA, at least one expert changed OSA diagnosis status based on the additional information provided by oximetry. Accordingly, multi-night OSA severity measurement provides clinically relevant information to help improve OSA diagnosis accuracy and clinical confidence.

Simplified multi-night home measurement technologies that use artificial intelligence and are deployed in the real-world setting have the potential to complement traditional polysomnography diagnoses of OSA or be used as a solitary diagnostic or screening tool in clear OSA/non-OSA cases. Despite the potential for higher diagnostic accuracy, we could not find any study that has deployed these technologies prospectively in a well-characterized clinical cohort. Therefore, the goal of this study was to assess the feasibility, acceptability and potential benefit of using simplified multi-night monitoring of OSA severity in a clinical cohort of patients with suspected OSA (*N* = 100). A secondary aim of this trial was to explore potential factors that may lead to misdiagnosis of OSA based on single-night polysomnography studies.

## Results

### Participant demographics

Out of the 100 recruited participants, four were excluded as they never set up their in-home OSA monitoring devices, four were excluded as they did not undergo polysomnography, see flow chart in supplementary Figure S1. The demographics of the 92 participants included in the final analysis are displayed in Table 1. Participants were middle-aged (mean ± SD; 53 ± 15 years old), overweight (BMI = 31.5 ± 7.5 kg/m^2^), and equally distributed between sexes, with 52% female and 48% male. Overall, based on single-night polysomnography-derived AHI, 66 participants (71%) had at least mild OSA (Table 1). Forty percent of participants with at least mild OSA were non-obese (BMI < 30 kg/m^2^). A total of 49 participants (53%) waited less than 3 months from the date of referral for an appointment with their sleep physician, 14 (15%) waited between 3 and 6 months, 8 (9%) between 6 and 12 months, and 21 (23%) more than 12 months. Study participants were mainly recruited from public hospitals (n = 75), followed by private practices (n = 17) and demographics were consistent across sites. There were 54 in-lab and 38 at-home polysomnography studies. Polysomnography was conducted temporally close to the in-home multi-night WSA testing in the vast majority of participants with only 7 participants completing polysomnography between 3 and 10 weeks prior to enrolment in this study.

**Table 1:** Participant demographics and co-morbidities stratified by obstructive sleep apnea (OSA) severity status as determined by polysomnography

	Overall	No OSA	Mild OSA	Moderate OSA	Severe OSA
*n*	92	26	21	18	27
Age, years	53.3 (14.9)	41.6 (14.3)	58.8 (13.7)	55.3 (10.1)	59.0 (13.1)
Sex: Female, *n* (%)	48 (52.2)	17 (65.4)	10 (47.6)	10 (55.6)	11 (40.7)
BMI, kg/m^2^	31.5 (7.5)	31.4 (9.5)	28.4 (3.9)	31.0 (7.1)	34.4 (7.0)
BMI above 30, *n* (%)	46 (50.0)	11 (42.3)	8 (38.1)	9 (50.0)	18 (66.7)
Systolic blood pressure, mmHg	127.3 (14.5)	121.2 (11.3)	122.5 (12.5)	134.1 (12.5)	132.2 (16.6)
Diastolic blood pressure, mmHg	83.8 (9.9)	81.2 (8.1)	79.3 (6.8)	87.7 (8.9)	87.3 (11.9)
Alcohol – AUDIT score	3.0 (2.4)	2.9 (2.0)	3.0 (2.6)	2.5 (1.9)	3.3 (2.9)
Smoker: yes, *n* (%)	35 (38.0)	6 (23.1)	10 (47.6)	10 (55.6)	9 (33.3)
Co-morbidities, *n* (%)					
Diabetes	9 (9.8)	2 (7.7)	2 (9.5)	1 (5.6)	4 (14.8)
Depression	33 (35.9)	13 (50.0)	3 (14.3)	7 (38.9)	10 (37.0)
Anxiety	36 (39.1)	16 (61.5)	4 (19.0)	5 (27.8)	11 (40.7)
Hypertension	22 (23.9)	1 (3.8)	6 (28.6)	7 (38.9)	8 (29.6)
Insomnia severity index	12.4 (6.1)	14.6 (5.4)	11.6 (7.0)	12.4 (5.8)	11.0 (5.7)
Epworth sleepiness scale score	7.2 (5.0)	8.0 (5.2)	6.9 (5.8)	7.9 (3.9)	6.3 (4.7)
PSG AHI, events/h	24.8 (29.2)	2.5 (1.6)	8.0 (2.4)	21.2 (4.0)	61.8 (28.6)

Data are presented as mean (SD) or *n* (%).

*PSG* polysomnography, BMI: body mass index, *AHI* apnea-hypopnea-index, *SOL* Sleep onset latency, *WASO* Wake after sleep onset.

### Feasibility and acceptability of in-home OSA monitoring

Eighty-five participants (92%) had at least 14 nights of valid AHI in their first months of in-home data collection. The median time between consent date when participants were provided with the home technologies, and the set-up of the WSA was 3 (IQR = 1.0–6.0) days. Most participants collected data every night in the first month (median = 30; IQR = 27–30 days) after initial set-up. Overall, in the first 3 months after informed consent, participants collected 80 (IQR = 69–86) nights of evaluable data. A total of 10% of measurements were unsuitable for AHI calculations because sleep duration was measured as <5 h.

A total of 76 of the 92 enroled participants completed the 3-month follow-up survey on technology adherence and perceptions. Sixty (79%) and sixty-one (80%) participants found that the device was mostly/very easy to use and convenient to use, respectively. Fifty-six participants (73%) reported checking the Withings mobile phone app more than four times a week. Fifty-four (71%) participants found the app mostly/very useful, 12 (16%) found it somewhat useful, and 10 (13%) found it slightly/not useful. Of the participants enroled in the study, 91 and 72 used the provided weight scale and blood pressure measurement device, respectively. Median usage of the weight scale and blood pressure cuffs in the first month was 6 (IQR = 3–9) and 4 (IQR = 3–70) measurements. A total of 65 (71%) and 52 (57%) of participants who used each device at least once met the measurement targets of four times per month for weight and blood pressure measures, respectively.

### Discordance between home-based and polysomnography-based OSA diagnosis

Six of the 92 study participants were already treated for OSA at consent date, three were treated before setting up the WSA, and five were treated within 30 days of using the WSA (see supplementary Figure S1). Sufficient WSA baseline data to compare in-home AHI vs. polysomnography AHI was therefore not available in these 14 participants and thus, they were excluded from this analysis. Data from the remaining 78 participants (52±16yo; BMI = 31 ± 8 kg/m^2^; 38 males/40 females) were analyzed included 44 in-lab and 34 at-home polysomnography studies. Out of the 78 participants, 29 cases of OSA and 33 without OSA were classified by both the WSA and polysomnography. Using polysomnography as the reference, WSA had a specificity of 85% and a sensitivity of 77% to detect OSA (AHI ≥ 15 events/h), and an overall F1-score to detect OSA of 80%. Sensitivity analyses restricted to in-home polysomnography (*N* = 34; specificity = 90%, sensitivity=86%, F1-score = 88% to detect OSA) and in-lab polysomnography (*N* = 44; specificity = 80%, sensitivity of 68%, F1-score =73) suggested similar performance. Excluding participants with polysomnography studies 3-10 weeks before commencement of the study also did not change the performance of the device in detecting OSA vs polysomnography (*N* = 74; specificity=83%, sensitivity=75%, F1-score = 79%). Using WSA as a reference, polysomnography had a specificity of 77% and a sensitivity of 85% to detect OSA, and an overall F1-score for detection of OSA of 80%. In other words, there were 10 participants classified as having OSA on WSA but not polysomnography, which would equate to a 26% misdiagnosis rate with polysomnography, assuming WSA as the gold-standard. Conversely, there were 6 participants with OSA on polysomnography but not WSA, which would equate to 15% misdiagnosis using WSA, assuming polysomnography as the gold standard. Similar misdiagnosis metrics were obtained using near-boundary double labelling (Table S1).

**Fig. 1:**
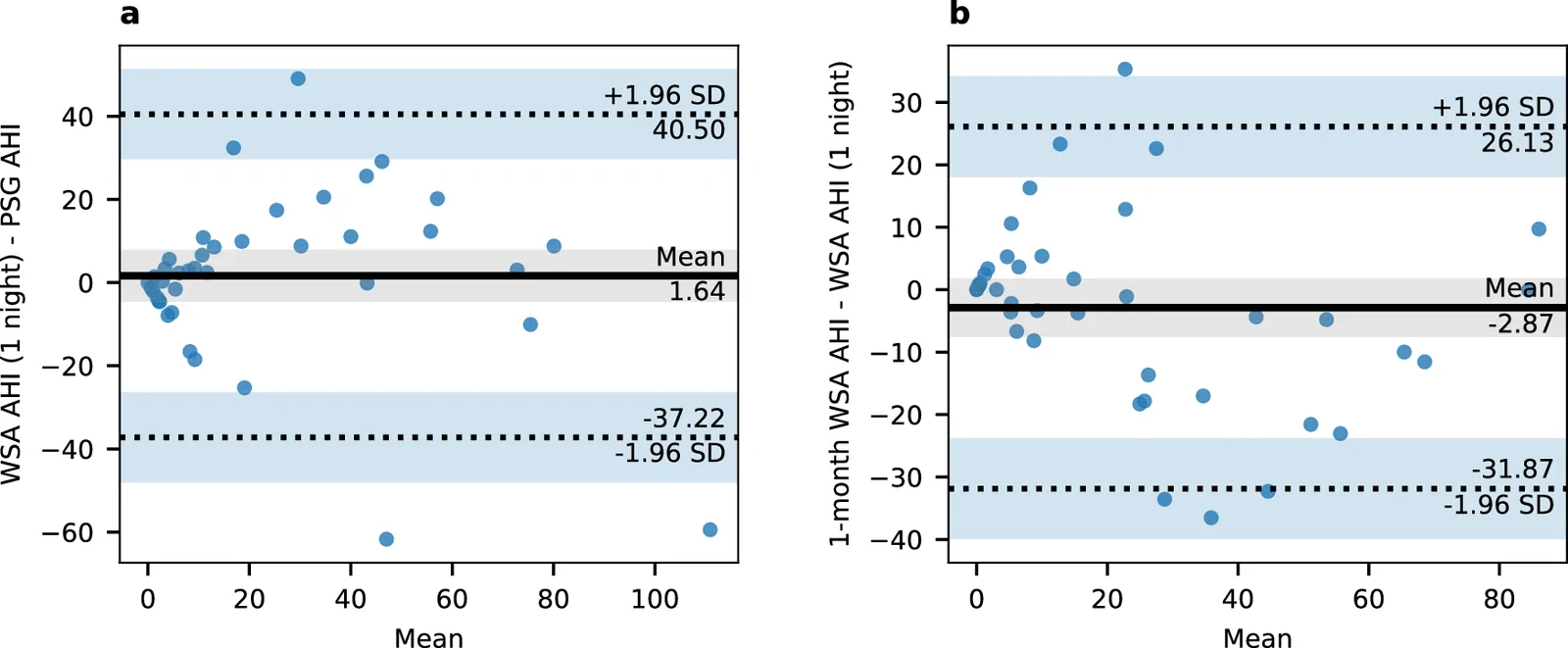
Bland-Altmann analysis between different recording modalities to estimate the apnea-hypopnea-index (AHI). **a** 1-night polysomnography AHI is compared to 1-night Withings Sleep Analyzer (WSA) estimated AHI. **b** 1-month WSA-estimated AHI is compared to 1-night WSA-estimated AHI.

In a subset of participants (*n* = 44), we collected 1-night WSA AHI on the same night as the polysomnography. In three participants, sleep duration was below 5 h. Thus, it was not possible to calculate the AHI on the WSA for these nights. In the remaining 41 participants (age = 52 ± 17 years old, BMI = 31 ± 7 kg/m^2^, 21 female/20 male), Bland-Altman analysis indicated no significant bias between 1-night polysomnography AHI and 1-night WSA AHI (see Fig. 1a; mean [95%CI]; 1.6 [−4.6, 7.9]). The 1-night WSA-estimated AHI had good agreement with contemporaneous in-laboratory polysomnography-derived AHI to classify moderate-to-severe OSA (86% sensitivity and 79% specificity to detect ≥15 events/h sleep; see confusion matrix in Table S2). Additionally, there was minimal bias between 1-month WSA AHI and 1-night WSA AHI (Fig. 1b; mean [95%CI]; −2.87 [−7.5, 1.8]). Agreement between 1-month WSA AHI and 1-night WSA AHI to classify moderate-to-severe OSA was also high (82% sensitivity and 90% specificity to detect ≥15 events/h sleep; see confusion matrix in Table S3).

### Discordance between home-monitoring and polysomnography-based OSA diagnosis

Adjusted for polysomnography AHI, participants with OSA on WSA but not polysomnography had higher night-to-night variability in OSA severity (mean difference [95%CI]; 9.53 [4.4, 14.6] events/h, *p* value < 0.001; Table 2). Furthermore, in unadjusted analysis and compared to participants with OSA on polysomnography but not on WSA, participants with OSA on WSA (but not on polysomnography) had lower sleep duration during the polysomnography night (−97 [−183, −11] min, *p* value = 0.027), and many had a tendency towards a higher proportion of sleep time spent supine (mean difference [95%CI]; 23.13 [−5.0, 51.3] %, *p* value = 0.106), and lower sleep efficiency (−11.8 [−24.4, 0.7]%, *p* value = 0.063). All participants had reduced sleep duration during the sleep study vs. in-home (Table 2), but the estimated difference was 100 min greater in participants “misclassified by polysomnography” (−100 [−200, 0] min). There were no significant differences in socio-demographic and clinical characteristics (e.g., ESS, ISI, FOSQ) between misclassification groups. Systolic blood pressure in participants with OSA on polysomnography but not on WSA tended to be lower (−14 [28, 0] mmHg). A total of 50% of the participants with OSA on polysomnography but not on WSA were shift-workers, while there were no shift workers among the participants with OSA on WSA but not on polysomnography (Table 2). Highlighted in supplementary material (Figures S2, S3 and S4) are 3 cases studies contextualizing the potential misdiagnosis of OSA by polysomnography due to poor sleep on the polysomnography night (Figure S2); “misdiagnosis” of OSA by WSA due to shift-work status and highly irregular sleep (Figure S3); and “misdiagnosis” of OSA by polysomnography due to very high night-to-night variability in OSA severity (Figure S4).

**Table 2:** Socio-demographics, clinical and sleep characteristics of participants classified by diagnosis status agreement between polysomnography (PSG) and Withings Sleep Analyzer (WSA)

	Agreement	Discordant		Agreement
	OSA WSA	OSA WSA	No-OSA WSA	No-OSA WSA
	OSA PSG	No-OSA PSG	OSA PSG	No-OSA PSG
*n*	29	10	6	33
Age, years	59.2 (11.6)	60.4 (14.6)	50.9 (18.5)	44.8 (15.8)
Sex: Female	13 (44.8%)	4 (40.0%)	3 (50.0%)	20 (60.6%)
BMI, kg/m^2^	33.3 (6.4)	29.5 (4.3)	31.9 (9.5)	30.1 (8.7)
AUDIT score	2.7 (2.3)	3.8 (3.1)	3.8 (3.5)	2.7 (1.9)
Systolic BP, mmHg	132.8 (16.3)	117.0 (9.4)	131.1 (10.5)	122.9 (12.7)
Diastolic BP, mmHg	87.9 (11.2)	79.0 (9.1)	83.3 (10.6)	80.8 (7.4)
ESS	7.1 (5.0)	7.8 (5.7)	5.5 (3.7)	7.6 (5.6)
ISI	11.2 (6.3)	11.4 (6.0)	12.8 (4.6)	13.6 (6.5)
Shift-worker: yes *n* (%)	0	0	3 (50%)	6 (18%)
WSAa				
AHI, events/hour	32.9 (24.5–51.1)	24.7 (20.2–27.1)	4.9 (3.6–9.7)	4.7 (0.8–8.7)
AHI variability^b^ events/hour	15.1 (8.5)	10.5 (4.6)	4.8 (2.9)	4.3 (3.0)
Sleep duration, min	414 (89)	444 (61)	441 (70)	414 (74)
Polysomnography (PSG)				
AHI, events/h	43.9 (25.4–71.3)	6.3 (5.2–8.1)	28.6 (22.1–33.8)	3.8 (1.6–6.2)
Supine AHI, events/h	48.8 (29.5–71.3)	22.9 (12.4- 35.2)	47.3 (26.6–57.7)	3.8 (0.3–8.7)
Non-supine AHI, events/hour	33.3 (15.9–71.3)	4.2 (2.1–5.9)	20.4 (16.1–30.7)	2.8 (1.7–4.9)
% sleep time in lateral position	60 (37–81)	90 (80–97)	60 (44–92)	62 (44–81)
Sleep duration, min	333 (87)	292 (73)	389 (69)	395 (85)
SOL, min	24 (20)	26 (29)	16 (8)	29 (41)
WASO, min	89 (52)	122 (41)	108 (83)	57 (47)
Sleep efficiency, %	73.1 (12.2)	64.2 (10.4)	76.0 (15.8)	79.4 (12.0)
PSG vs. home sleep duration	−73 (−143–−13)	−161 (−208–−97)	−47 (−63–−41)	−23 (−80–40)

Data are presented as mean (SD), *n* (%) or median (IQR).

*PSG* polysomnography, *WSA* Withings sleep analyzer, *BMI* body mass index, *BP* blood pressure, *ESS* Epworth sleepiness scale, *ISI* Insomnia severity index, *AHI* apnea-hypopnea-index, *SOL* Sleep onset latency, *WASO* Wake after sleep onset.

^a^Calculated using baseline period of 30 days on either side of the sleep study.

^b^Root-mean-squared of successive difference.

## Discussion

In this prospective observational trial of 100 participants with suspected OSA, we show that nightly monitoring of OSA severity using zero-burden, novel under-mattress technology driven by artificial intelligence algorithms is feasible and well tolerated. Weight and blood pressure measurement technologies were also acceptable to most participants. Most people (92%) met the target number of measurements needed for an estimation of OSA severity at baseline, and 70–80% found the devices and apps mostly useful. We also showed that multi-night simplified monitoring of OSA in the real-world setting provides an accurate (sensitivity and specificity ~80%) estimation of OSA status compared to polysomnography. Taken together, these results demonstrate the feasibility and acceptability of multi-night zero-burden technologies in the clinical management of OSA and suggest that such devices may aid in the accurate diagnosis and monitoring of OSA and its key consequences.

Overall, the acceptance of the home monitoring technologies was high, despite little intervention to ensure adherence. Ad-hoc qualitative feedback suggested that, while most participants had no difficulty setting up the under-mattress technology, some participants had technical difficulties regarding Wi-Fi connectivity issues (often due to modem distance or unfamiliarity with Wi-Fi passwords). Less tech-savvy participants also struggled to navigate the app. Adherence for daytime weight and blood pressure measurements was similar to app-based measurement adherence in other chronic health conditions, while, as expected, the success rate of nightly sleep measurements was far higher. Indeed, this is likely due to the “set-and-forget”, zero-burden nature of the under-mattress device and supports existing evidence that the ease of use is a critical factor in adherence to similar technologies^43,44^. Regarding the more moderate daytime measurement adherence, a large body of evidence has shown that prompting is useful and acceptable to increase treatment and medication adherence^45^. However, factors such as perceived prompt urgency or mental load can significantly impact effectiveness^46^. Consequently, more personalised and specific prompts may be useful in the future to maximise adherence to weight and blood pressure measurements. In addition, ad-hoc qualitative feedback suggested that some participants had difficulty with self-administration of the blood pressure device, specifically around cuff-size, with this likely a barrier to adherence.

Overall, specificity was 85%, and sensitivity was 77% to classify moderate-to-severe OSA compared to in-home based WSA-estimated OSA vs. gold standard polysomnography. Similarly, we found that the 1-night WSA-estimated AHI had good agreement with contemporaneous polysomnography-derived AHI to classify moderate-to-severe OSA (86% sensitivity and 79% specificity). These accuracy numbers are in accordance with previously published validation studies^2,38,47^, and similar in accuracy to other FDA-cleared wearables and nearables^40^. Our secondary analyses that explored the discordance between single-night polysomnography and multi-night under-mattress sensor suggest a need for both approaches in the accurate diagnosis of OSA. Poor sleep on polysomnography was correlated with a higher likelihood of underestimating OSA severity compared to in-home monitoring. Conversely, more variable OSA severity was associated with a higher likelihood of detecting OSA using multi-night measurements compared to polysomnography. Irregular sleep patterns, such as those experienced during shiftwork (see case study 2), may be problematic for OSA severity estimation with simplified monitoring. That said, the relatively small sample size in our prospective study precluded investigation into the accuracy of the device to estimate misclassification of OSA severity categories (mild, moderate or severe). We were also underpowered to detect differences between discordant groups. Therefore, our results on the factors driving misclassification of OSA severity (e.g., poor sleep, shift-work) need to be interpreted with caution. Similarly, we could not investigate many potential physiological and behavioural mechanisms of OSA that may explain OSA variability, such as endotypes or proportion of flow-restricted breathing and snoring^12,48–50^, alcohol and food intake^22,32^, or sleeping position, which is likely to be one of the largest drivers of night-to-night variability in OSA^22–24^. Nonetheless, minimal time spent supine during polysomnography was associated with discordant AHI findings in the home for many participants. Future work is required to explore how these drivers of OSA variability impact the single- and multi-night classification of OSA.

The device used in this study has some limitations that may preclude its use as a stand-alone diagnostic modality at the time of writing. Firstly, the lack of oximetry is problematic, given that hypoxia is likely to be one of the main mechanisms linking OSA to poor cardiovascular health^51,52^. Ventilatory burden, a measure of flow limitation severity for hypopnea and apnea events, could be a plausible surrogate for oximetry given the high correlation between ventilatory and hypoxic burden^53,54^. However, this is not yet possible with a non-contact sleep monitoring device. Additionally, at the time of this writing, the device is not able to measure body position. Thus, supine-predominant OSA cannot be detected. The device cannot differentiate between hypopneas, apneas and central events, which may be important for appropriate treatment^55^. The lack of these physiological parameters may meaningfully restrict the clinical applicability of the device as a stand-alone diagnostic tool, particularly in risk stratification and phenotyping of OSA. Finally, device performance to estimate sleep duration and sleep staging appears to be reduced in those with poor sleep and in people with irregular sleep schedules (e.g., daytime naps)^56^. These may be particularly problematic in some subtypes of OSA with poorer sleep, such as insomnia and OSA overlap, which are associated with worse health outcomes^57,58^. Of note, most of these limitations are engineering problems that may be solvable with future technology and signal processing development. In the meantime, to investigate the key mechanisms that drive high night-to-night variability in OSA severity for which changes in head, neck and body position are likely to be influential^59–62^, the addition of home-monitoring technology beyond an under-mattress sensor to capture these key variables would be insightful^22^.

Despite these potential limitations, multi-night OSA estimates provide useful complementary clinical insight to current single-night measurements. Indeed, the current device provides invaluable information that may contextualise OSA diagnosis and help guide treatment. Multi-night measurements of OSA using simplified technologies like the one described here could be used as a replacement for questionnaires for effective and accurate screening of OSA before a sleep study. Multi-night measurements of OSA may also help readily detect people with highly variable OSA severity who are at an increased likelihood of being misdiagnosed (exemplified in one of our case studies) and may be at increased risk of poor cardiovascular health^16–18,20,21^. Beyond screening and diagnosis, multi-night zero-burden OSA assessment could provide estimates of residual AHI and treatment effectiveness^63^, adding substantial value over conventional treatment adherence metrics, which do not account for periods of non-use. As this was an observational feasibility study, investigation of potential changes to patient clinical care due to multi-night monitoring was out of scope. Prospective appropriately sized randomised controlled trials are needed to determine the most appropriate and effective technologies for OSA diagnosis. Potential risks versus benefits, patient perspectives, and cost-effectiveness of these novel approaches should be carefully considered.

In conclusion, we demonstrate that multi-night monitoring of OSA severity using an artificial intelligence-enabled, zero-burden, under-mattress sensor in the real-world setting is acceptable, feasible, and accurate to detect OSA status. Our study further supports the unique and important insights that multi-night non-invasive monitoring can yield, especially in reducing misdiagnosis rates for people who experience poor sleep during polysomnography or who have highly variable OSA severity.

## Methods

### Study design

This study was a prospective observational clinical cohort study of 100 participants with suspected OSA. Consenting participants were provided with technologies to monitor their sleep, blood pressure, weight and report general daytime impairment throughout the study period (3 months). Participants underwent usual clinical care, including gold-standard polysomnography as required, in parallel with this research study. Informed consent to participate in the study was obtained from all participants, and this protocol was approved by the Southern Adelaide Clinical Human Research Ethics Committee (Project number: LNR/22/SAC/78). Clinical trial number: not applicable.

### Participants

Potential participants were consecutively recruited during routine attendance at sleep medicine clinics, both private and public, in South Australia, between September 2022 and June 2024. There were six recruitments sites: three public sleep clinics and three private practices. Specifically, after referral to a specialist sleep clinic for possible OSA, consenting participants were given education on home monitoring technologies, and asked to complete baseline measurements and surveys. Participants were required to be at least 18 years old and were required to have and be able to operate home WiFi and a compatible smartphone.

### Zero-burden, AI-enabled monitoring technologies

Participants were provided with a Withings Sleep Analyser (WSA), an FDA/TGA-cleared (FDA 510(k) number: K231667) non-wearable sleep monitoring device, to be placed under their mattress to continuously monitor AHI and sleep stages throughout the study period. The WSA-estimated AHI has good agreement with in-laboratory polysomnography-derived AHI, with ~80% sensitivity and specificity to classify moderate-to-severe OSA across multiple validation studies^2,38,47^, similar to other FDA-cleared wearables and nearables^40^. An initial validation study reported high accuracy for mild OSA classification (AHI ≥ 5 events/h, sensitivity = 89%, specificity = 75%), moderate-to-severe OSA classification (AHI ≥ 15 events/h, sensitivity = 88%, specificity = 88%), and severe OSA classification (AHI ≥ 30 events/h, sensitivity = 86%, specificity = 91%)^38^. When evaluated as a continuous variable, WSA-estimated AHI has minimal bias (mean [95% limits of agreement]: 1.6 [ − 0.7, 4.1]^38^ and −1.38 [2] events/h), and moderate-to-strong correlation with polysomnography-derived AHI (*r*^2^ = 0.59^64^; *r* = 0.40^38^; *r* = 0.69^47^, *r* = 0.70^2^). Validation studies have evaluated performance using polysomnography scoring based on the AASM 2012 criteria, including hypopnea definitions requiring a ≥ 30% airflow reduction with either ≥3% oxygen desaturation or an arousal^65^. The AHI cannot be reliably calculated for nights with a sleep duration of less than 5h^38^. Multiple independent studies in over 600 participants have validated WSA-estimated sleep duration against polysomnography^56,66,67^. Similar to other wearable/nearable devices^68,69^, the WAS tends to overestimate sleep duration by ~20-40 min versus polysomnography. Participants were additionally provided with an FDA-cleared Withings Blood pressure monitor and Withings Body Cardio scale. All devices were connected to the cloud via the participants’ home Wi-Fi using de-identified study accounts.

### Baseline assessment

After initial consent, study personnel collected baseline measurements of blood pressure, height, weight, bioelectrical impendence, participant-reported medical history and validated sleep questionnaires. Each participant was provided with the monitoring technologies described above and instructed on in-home installation of the devices. Baseline sleep and health questionnaires administered included the Epworth Sleepiness Scale (ESS), Karolinska Sleepiness Scale (KSS), Insomnia Severity Index (ISI), Functional Outcomes of Sleep Questionnaire (FOSQ-10), and Quality of Life Questionnaire (EQ-5D-5L). In addition, participants were also asked to complete a custom questionnaire regarding preferences, ability, and feelings surrounding interaction with health technology.

### Polysomnography

Gold-standard in-laboratory (level 1) or home (level 2) polysomnography studies were conducted as part of participants’ usual care at the discretion of their sleep physician. Some participant sleep studies had been conducted a few months prior to enrolment, in which case summary sleep study statistics from sleep reports were accessed, where possible. Studies were scored according to the American Academy of Sleep Medicine current recommended scoring guidelines^70^. Hypopneas were scored as a reduction of flow above 30% of baseline and a 3% desaturation and/or an arousal^70^. OSA severity was based on predefined AHI cut-off scores (<5 = no OSA, ≥5 and <15 = mild, ≥15 and <30 = moderate and ≥30 events/h = severe OSA)^70^.

### Follow-up assessment

Participants were asked to take daytime blood pressure measurements and body weight recordings weekly for the duration of the study (3 months). Two email or text reminder messages were sent to participants in case of missed blood pressure and weight measurements. At 3 months, participants completed a follow-up survey mirroring the baseline questionnaire. Participants reported any commencement or changes in OSA treatment, medications, or other medical treatment in the 3-months prior. In addition, participants reported willingness to use each of the devices, their ease of use, and how often they interacted with the Withings mobile application via 5-point Likert scale. Participants were sent email reminders to complete the questionnaires if their responses were not received a week after they were due for completion.

### Primary outcomes

Our primary outcome was feasibility of the use of novel in-home technology to measure OSA severity, defined as the number of participants who had ≥14 successful sleep recordings in the first month of recording. This 14-day threshold was selected given it has previously been shown to be the optimal number of nights for an OSA diagnosis to minimize misclassification^2,17^. Secondary feasibility outcomes included the median (IQR) number of days from consent date to setup the prescribed technology, and the number of recordings in the first three months of data collection. Secondary feasibility outcomes were calculated for the OSA monitoring device and the daytime monitoring (blood pressure and weight). Blood pressure and weight feasibility outcomes were operationalised as at least four recordings per month in the first month.

### Secondary outcomes

The accuracy of the WSA to detect OSA in our clinical cohort, as compared with current gold-standard of single-night polysomnography, was explored in secondary analyses. In most cases, where participants had no difficulty setting up their WSA, we used 60 days centred around the sleep study (30 days before and/or 30 days after) as the “baseline” period. In cases where the sleep study was completed before the WSA was set up, the first 30 days of in-home monitoring were instead used as the “baseline” period. Average AHI was calculated over the baseline period for each participant, and we compared diagnostic status based on WSA-estimated vs. polysomnography-derived AHI to classify moderate-to-severe OSA ( ≥ 15 events/h).

### Statistical analyses

Descriptive analysis of devices usage, mean (SD) and median (IQR) and n (%), were used to summarize our primary outcomes. We used specificity, sensitivity and F1-score to evaluate the agreement between polysomnography and WSA-based diagnosis of OSA. The term “misclassified” is used bidirectionally in our manuscript, and we assumed that both polysomnography and the WSA could “misclassify OSA” compared to the other device. Since we did not make assumptions about the best diagnostic modalities, we calculated the metrics separately using WSA or polysomnography as the gold standard diagnostic. In secondary analyses, we investigated unadjusted associations of polysomnographic, demographic, and clinical characteristics between participants classified as having OSA on WSA but not on polysomnography (or vice-versa) using linear regression with diagnostic agreement status (categorical) as the exposure variable. In some cases, these models were adjusted for OSA severity as determined by polysomnography.

### Sensitivity analysis: near boundary double labelling

Inter-scorer variability in scoring of apneas, hypopneas and arousals^71,72^ invariably leads to variability in OSA diagnostic status. Polysomnography-derived AHI close to diagnostic cut-offs should therefore be treated with caution, especially when comparing diagnostic modalities or devices. An approach to minimize this effect is to use near boundary double labelling techniques^37^, whereby participants close to the diagnostic cut-offs are counted as both having and not having the disease. These boundary cut-offs for AHI 15 events/hour were determined empirically in a previous study as 12.4-17.4 events/h^37^. In this sensitivity analysis, misclassification rates between polysomnography and WSA were calculated using near boundary double labelling techniques, where any participants with polysomnography AHI between the boundary cut-offs was considered to have both non-OSA and OSA.

## Supplementary information

Supplementary Information

## Data Availability

The datasets generated and/or analyzed during the current study are not publicly available due to privacy and ethical reasons. Deidentified data that support the findings of this study, including individual data, are available from the corresponding author upon request subject to ethical approval.

## Code availability

No custom code was developed as part of this study, and underlying code for statistical analysis is not publicly available but may be made available to qualified researchers on reasonable request from the corresponding author (bastien.lechat@flinders.edu.au).
