# Supplementary material for: Zero burden multi night monitoring with AI enabled technology reduces obstructive sleep apnea misdiagnosis
Source: NPJ Digit Med. 2026 Jun 16;9:753. doi: 10.1038/s41746-026-02914-w (PMC13635168; doi:10.1038/s41746-026-02914-w)
Supplement: Supplementary file 1 [file 41746_2026_2914_MOESM1_ESM.pdf]

Supplementary: Zero burden multi night monitoring with AI enabled technology reduces obstructive sleep apnea misdiagnosis

Bastien Lechat[1], Simon Proctor[1], Jack Manners[1], Kelly A. Loffler[2], Alison Leviton[1], Barbara Toson[1], Daniel Vena[3], Andrew Vakulin[1], Josh Fitton[1], Robert J. Adams[1], Sutapa Mukherjee[1], Danny J Eckert[1]

[1] Flinders Health and Medical Research Institute (FHMRI) Sleep Health and Adelaide Institute for Sleep Health,Flinders University, Adelaide, Australia
[2] Flinders Clinical Trials, Flinders University, Bedford Park, Australia
[3] Division of Sleep and Circadian Disorders, Brigham and Women's Hospital and Harvard Medical School, Boston, MA, USA

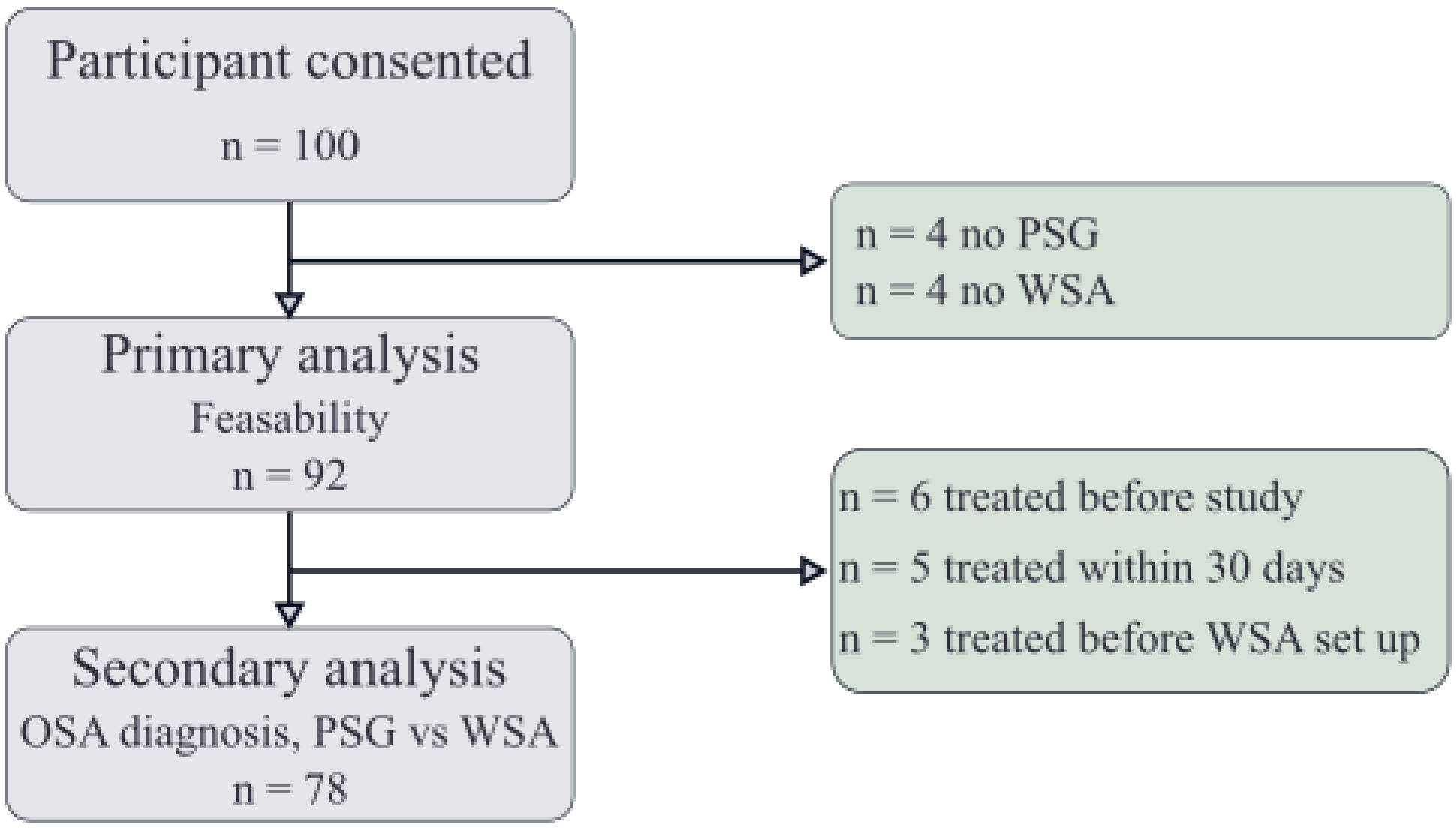

Figure S1: *Flowchart of participants included in the primary and secondary analyses in this study.*

Sensitivity analysis: near boundary double labelling

There were 5 participants in the near boundary zone with an AHI between 12.4 and 17.4 events/h during the polysomnography study. Further accounting for these participants as having both OSA and non-OSA did not change the main classification metrics. Indeed, compared to the main analysis, the number of participants with OSA on polysomnography but not WSA remained similar (n=6). Participants with OSA on WSA but not on polysomnography increased from 10 to 13. Overall, F1-score for the classification of OSA was 0.77 both when polysomnography or WSA was used as the reference. Using polysomnography as the reference, WSA had a specificity of 85% and a sensitivity of 72% in detecting OSA. Using WSA as the reference, polysomnography had a specificity of 72% and a sensitivity of 85% in detecting OSA.

Table S1: *Classification metrics for WSA vs polysomnography based of OSA diagnosis (apnoea-hypopnea-index ≥ 15 events/h) using near boundary double labelling techniques.*

		**Main analysis**			**Sensitivity**	
		Polysomnography			Polysomnography	
		**No OSA**	**OSA**		**No OSA**	**OSA**
Withings Sleep Analyzer	**No OSA**	33	6	**No OSA**	33	6
	**OSA**	10	29	**OSA**	13	31

Table S2: *Classification metrics for 1-night WSA vs polysomnography based OSA diagnosis (apnoea-hypopnea-index ≥ 15 events/h).*

		**Main analysis**	
		Polysomnography	
		**No OSA**	**OSA**
1-night Withings Sleep Analyzer	**No OSA**	14	3
	**OSA**	5	19

Table S3: *Classification metrics for 1-month WSA vs 1-night WSA based OSA diagnosis (apnoea-hypopnea-index ≥ 15 events/h).*

		**Main analysis**	
		Polysomnography	
		**No OSA**	**OSA**
1-month Withings Sleep Analyzer	**No OSA**	17	4
	**OSA**	2	18

<u>Case studies</u>

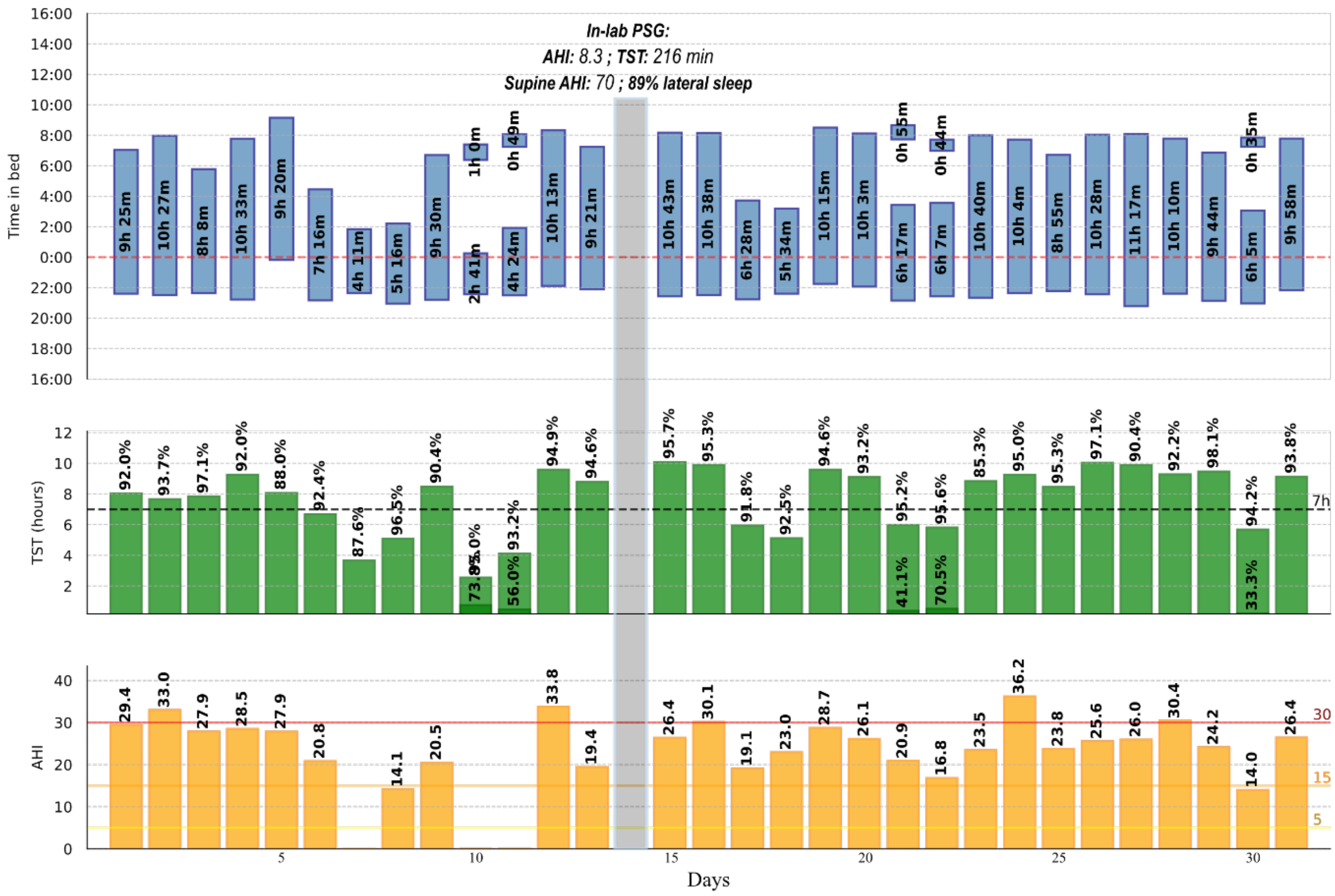

Figure S2: *Potential misdiagnosis by polysomnography due to poor sleep during study night. The participant slept 3h 36min during polysomnography, spent 89% of their sleep in lateral position. Overall, the AHI was 8.3 events/h, but supine AHI (70 events/h; 24 min supine) and REM AHI (44.1 events/h; 34 min REM sleep) were much higher. Short sleep as experienced during the sleep study night is uncommon in the home but happens on some nights. Bedtime is consistent on most nights (between 9 and 10pm), but early morning/middle of the night awakening happens in a few nights (often followed by a nap). These sleep patterns correlate with the insomnia severity index (17) including reporting severe difficulties staying asleep and waking up too early.*

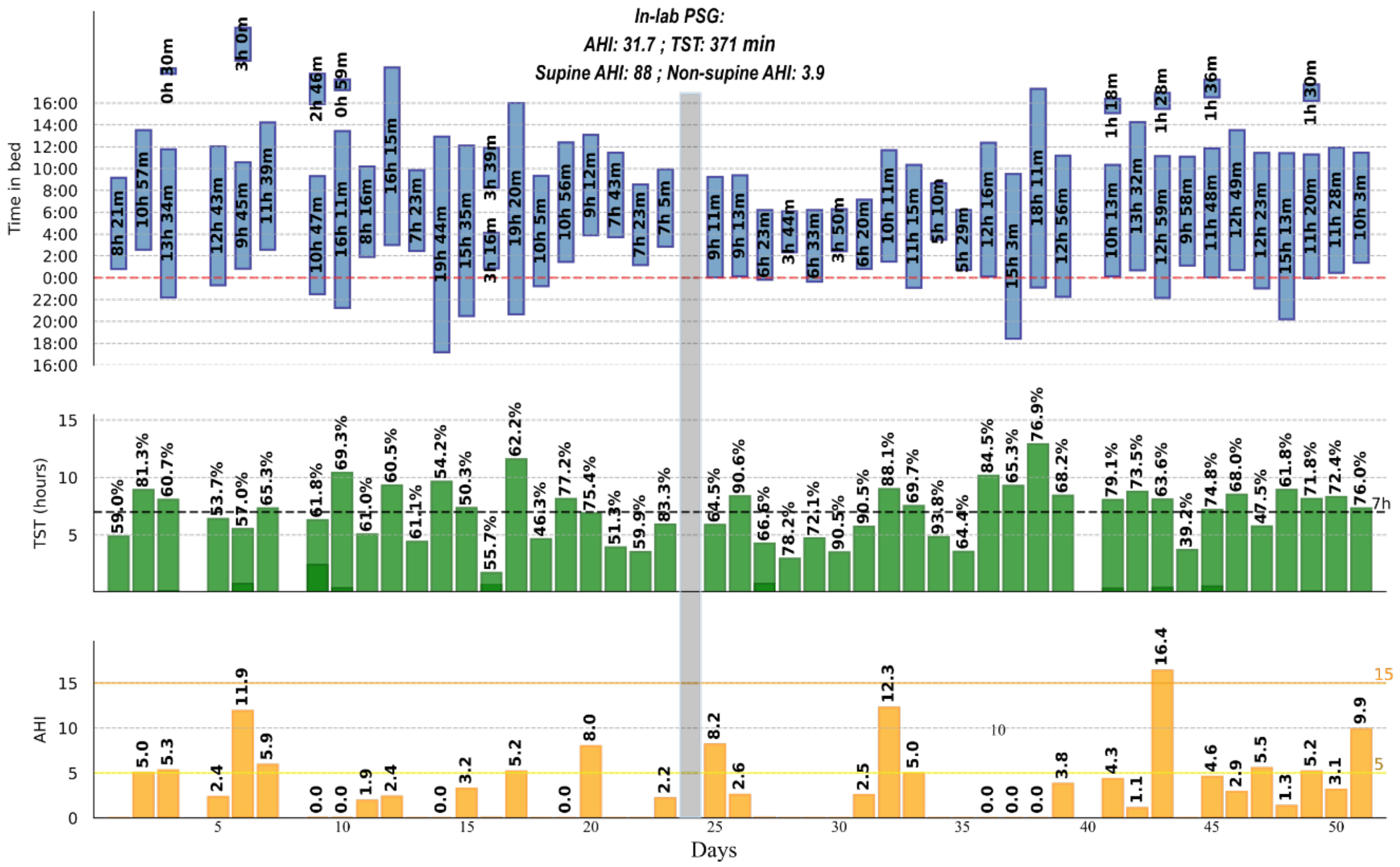

Figure S3: *Potential misdiagnosis by WSA due to shiftwork and highly irregular sleeping patterns. The participant slept 6h 11min during polysomnography. Overall, the AHI was 31.7 events/h, but supine AHI (88 events/h) was much higher than non-supine AHI (3.9 events/h). In the home, sleep patterns are highly irregular, with some time in bed periods of more than 12 hours, bedtime irregular (between 8pm and 4 or 5am). This is not surprising since the participant reported doing shiftwork. In the home, AHI varies from 1 to 16.4 events/hs, but many nights are unsuitable to calculate AHI since sleep duration is often below 5h.*

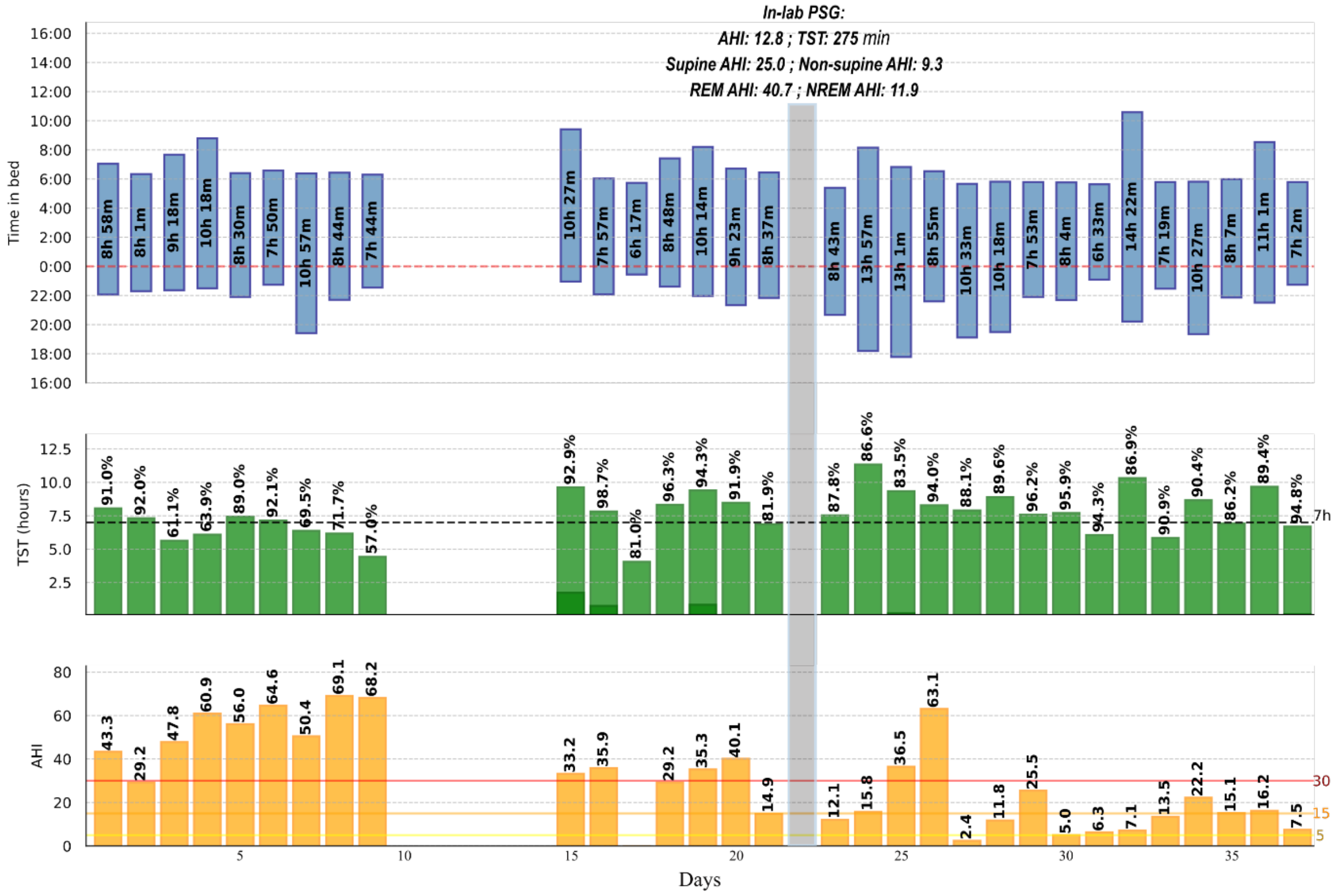

Figure S4: *Potential misdiagnosis by polysomnography due to high night-to-night variability in OSA severity. The participant slept 4h 35min during polysomnography. Overall, the AHI was 12.8 events/h, with small differences between supine AHI (25.0 events/h) and non-supine AHI (9.3 events/h). In the home, sleep patterns are mostly regular. However, OSA severity night-to-night is highly irregular and range from 2.4 events/hours to ~70 events/h. Most nights have an AHI above 15 or 30 events/h.*

STARD CHECKLIST

Section & Topic	No	Item	Reported on page #
**TITLE OR ABSTRACT**			
	**1**	Identification as a study of diagnostic accuracy using at least one measure of accuracy<br>(such as sensitivity, specificity, predictive values, or AUC)	2 (abstract)
**ABSTRACT**			
	**2**	Structured summary of study design, methods, results, and conclusions<br>(for specific guidance, see STARD for Abstracts)	Feasibility trial
**INTRODUCTION**			
	**3**	Scientific and clinical background, including the intended use and clinical role of the index test	2 and 3
	**4**	Study objectives and hypotheses	3
**METHODS**			
Study design	**5**	Whether data collection was planned before the index test and reference standard<br>were performed (prospective study) or after (retrospective study)	3
Participants	**6**	Eligibility criteria	3 and 4
	**7**	On what basis potentially eligible participants were identified<br>(such as symptoms, results from previous tests, inclusion in registry)	3 and 4
	**8**	Where and when potentially eligible participants were identified (setting, location and dates)	3 and 4
	**9**	Whether participants formed a consecutive, random or convenience series	4
Test methods	**10a**	Index test, in sufficient detail to allow replication	5 and 6
	**10b**	Reference standard, in sufficient detail to allow replication	6 and 7
	**11**	Rationale for choosing the reference standard (if alternatives exist)	8
	**12a**	Definition of and rationale for test positivity cut-offs or result categories<br>of the index test, distinguishing pre-specified from exploratory	7 and 8
	**12b**	Definition of and rationale for test positivity cut-offs or result categories<br>of the reference standard, distinguishing pre-specified from exploratory	
	**13a**	Whether clinical information and reference standard results were available<br>to the performers/readers of the index test	n.a.
	**13b**	Whether clinical information and index test results were available<br>to the assessors of the reference standard	n.a.
Analysis	**14**	Methods for estimating or comparing measures of diagnostic accuracy	7
	**15**	How indeterminate index test or reference standard results were handled	7<br>near-boundary labelling
	**16**	How missing data on the index test and reference standard were handled	6 and 7
	**17**	Any analyses of variability in diagnostic accuracy, distinguishing pre-specified from exploratory	8
	**18**	Intended sample size and how it was determined	n.a. – Feasibility study
**RESULTS**			
Participants	**19**	Flow of participants, using a diagram	8 – Figure S1
	**20**	Baseline demographic and clinical characteristics of participants	8
	**21a**	Distribution of severity of disease in those with the target condition	8
	**21b**	Distribution of alternative diagnoses in those without the target condition	8
	**22**	Time interval and any clinical interventions between index test and reference standard	8 and 9
Test results	**23**	Cross tabulation of the index test results (or their distribution)<br>by the results of the reference standard	10
	**24**	Estimates of diagnostic accuracy and their precision (such as 95% confidence intervals)	10
	**25**	Any adverse events from performing the index test or the reference standard	n.a.
**DISCUSSION**			
	**26**	Study limitations, including sources of potential bias, statistical uncertainty, and generalisability	13 and 14
	**27**	Implications for practice, including the intended use and clinical role of the index test	13 and 14

**OTHER INFORMATION**			
	**28**	Registration number and name of registry	n.a. Feasibility study
	**29**	Where the full study protocol can be accessed	n.a. Feasibility study
	**30**	Sources of funding and other support; role of funders	15
